# The *C. elegans* pharyngeal basement membrane has a high affinity for binding agrin

**DOI:** 10.17912/micropub.biology.002449

**Published:** 2026-10-21

**Authors:** C. Teresa Ibeanu, Sandhya Srinivasan, Qiuyi Chi, David R. Sherwood

**Affiliations:** 1 Biology, Duke University, Durham, NC, United States

## Abstract

Basement membranes (BMs) are dense, sheet-like extracellular matrices that surround animal tissues and provide mechanical and signaling functions. Tissue BMs are compositionally unique, with the presence and abundance of numerous components tailored to meet tissue-specific needs. How BMs establish and maintain distinct compositions remains poorly understood. Here, we investigate how the BM protein agrin is uniquely concentrated in the *C. elegans* pharyngeal BM. Through tissue-specific expression and live imaging, our results suggest that a combination of pharyngeal-specific expression and high BM affinity for binding agrin ensures the unique enrichment of agrin in the pharyngeal BM.

**Figure 1. Agrin preferentially localizes to the pharyngeal BM:** (A) Schematic representation of the three separate constructs used to drive *agr-1 ::mScarlet-I* (agrin) expression. The *agr-1* gene encoding *C. elegans* agrin tagged with mScarlet-I was expressed in the body wall muscle using the *myo-3* promoter (top; depicted in pink), in the intestine using the *pept-1* promoter (middle; depicted in yellow) and pan-neuronally, including the nerve ring using the *rab-3* promoter (bottom; depicted in green). All three constructs used the *unc-* 54 3' UTR. (B) Schematic diagram of an L4 larva depicting the location of the analyzed tissues and their surrounding BMs (dashed lines). (C) Boxplots show the average fluorescence intensity of extracellular AGR-1 mScarlet-I signal (see methods; median of data denoted by horizontal line within each box; *n* > 5 animals each). (D) Single z-slice confocal images show the localization of endogenous AGR-1 protein tagged with mNeonGreen (mNG; first column) and AGR-1 attached to mScarlet-I expressed in the body wall muscle (second column), intestine (third column), and neurons (last column) in the BMs surrounding the pharynx, body wall muscle, intestine and nerve ring. Yellow arrows indicate AGR-1 localization to the BM. The scale bar is 10 μm. (E) Single z-slice confocal images of the pharyngeal tissue show internalized puncta of endogenous AGR-1 ::mNG (first column) and AGR-1 ::mScarlet-I expressed in the body wall muscle (second column), intestine (third column), and neurons (last column). The scale bar is 10 μm. (F) Table shows average fluorescence intensity of AGR-1 fluorescence in the BMs surrounding the pharynx, body wall muscle, intestine and nerve ring. The site of expression is specified in the last four columns of the table, with the first column shows average fluorescence values of endogenous AGR-1 ::mNG (highlighted in green). The last three columns show average fluorescence values of AGR-1 ::mScarlet-I (highlighted in red) expressed from the body wall muscle, intestine and neurons (n = 10 animals examined for each). Tissue BMs where agrin was not detected are denoted as ND (“not detected”) in BM. (G) Boxplots show the average number of AGR-1 puncta localized to a 50 μm ^2^ region (see methods) within the anterior bulb of the pharynx (median of data denoted by horizontal line within each box; *n* = 5 animals each; **P < 0.01, ***P < 0.001, ANOVA with Tukey's multiple comparisons).

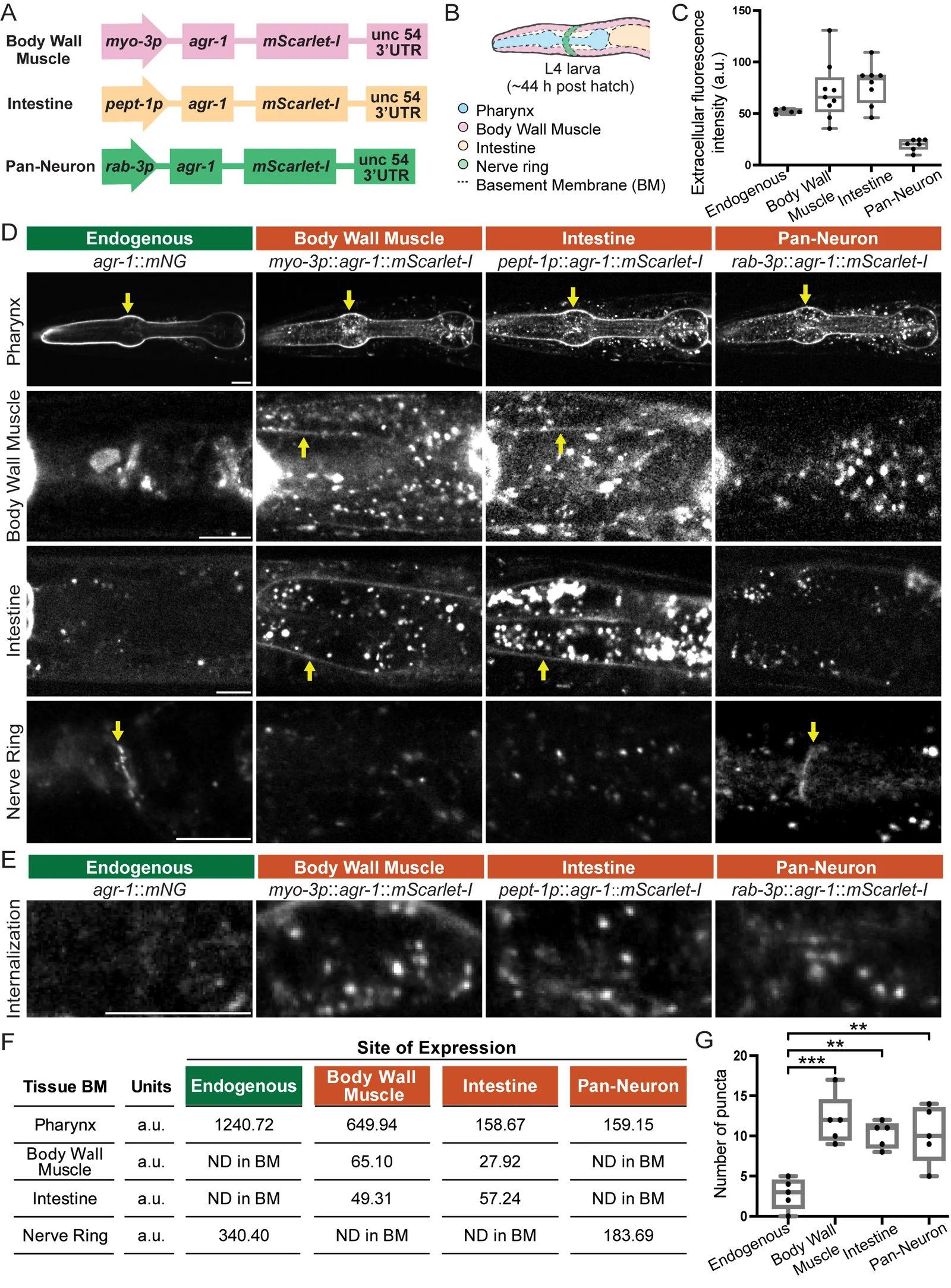



## Description

Basement membranes (BMs) are thin, dense extracellular matrices that surround most tissues to provide structural support, barrier function, and signaling platforms for a diverse range of cellular behaviors (Lennon & Sherwood, 2025). BMs are largely composed of two self-associating networks of secreted laminin and type IV collagen but also contain over 150 other secreted proteins in vertebrates and over 75 in *C. elegans* (Jayadev et al., 2022). BMs surrounding different tissues vary in the presence and levels of individual BM components, which is thought to support the needs of different tissues (Jayadev et al., 2019; Jayadev et al., 2022; Randles et al., 2017; Sekiguchi & Yamada, 2018; Sherwood, 2021). Basement membrane composition and organization are tightly regulated, and disruption of BM make-up impairs tissue morphogenesis, organogenesis, and tissue function (Jayadev & Sherwood, 2017; Sekiguchi & Yamada, 2018; Topfer, 2023). Furthermore, alterations in BM composition and organization contribute to the pathogenesis of numerous diseases, including cancer and kidney disease (Pozzi et al., 2017).

Tissue-specific BM composition is thought to be governed by high affinity binding of BM molecules to pre-existing BM proteins, local proteolytic processing of BM proteins to create high affinity interactions, and the binding of BM components to cell surface receptor (Clay & Sherwood, 2015; Gianakas et al., 2023; Isabella & Horne-Badovinac, 2016; Jayadev et al., 2019). BM proteins are in many cases contributed locally by the cells in direct contact with the BM (Clay & Sherwood, 2015; Haigo & Bilder, 2011; Isabella & Horne-Badovinac, 2016; Zajac & Horne-Badovinac, 2022). Live imaging, site of expression, and localization studies in *C. elegans* and *Drosophila* have also revealed that several BM components, including laminin and type IV collagen, can be secreted from distant cellular sources, diffuse through the extracellular fluids, and then be incorporated into BMs (Clay & Sherwood, 2015; Graham et al., 1997; Matsubayashi et al., 2017; Pastor-Pareja & Xu, 2011). Our understanding of how distinct BMs are built, however, is still limited to only a handful of BM components and the mechanisms governing the specific incorporation of most BM proteins remain unexplored.

Agrin is a large heparan sulfate proteoglycan and a component of many BMs (Denzer et al., 1996; Iozzo et al., 2009; Tatrai et al., 2006). Agrin may contribute to BM stability by linking laminin networks to the cell-surface receptor α-dystroglycan (McKee et al., 2009; Moll et al., 2001; Yurchenco, 2011). In addition **,** agrin can bind members of the TGF-β/BMP family and modulate their signaling activity (Banyai et al., 2010). Notably, agrin is selectively concentrated in specific human and mice BMs, such as the kidney glomerular BM and lung alveolar BM (Groffen, Buskens, et al., 1998; Groffen, Ruegg, et al., 1998). *C. elegans* contains a single agrin ortholog, AGR-1 , with a domain organization resembling that of vertebrate agrin (Hrus et al., 2007). Expression data, antibody localization and analysis of a functional genomic, in frame knock-in of mNeonGreen (AGR-1::mNG), has shown that the *C. elegans* agrin is selectively enriched in the BM surrounding the larval pharynx (muscular feeding organ) and expressed in the pharyngeal epithelial cells. It is also present at lower levels in the BM surrounding the nerve ring, a U-shaped axon bundle surrounding the isthmus of the pharynx and expressed in IL1 neurons that contribute to the nerve ring (Altun, 2017; Hrus et al., 2007; Keeley et al., 2020). These data suggest that *C. elegans* agrin might require local synthesis and deposition to enrich in specific BMs. Whether agrin-rich BMs bind agrin with a high affinity and can incorporate agrin when secreted from distant sources is unknown.

To determine if agrin can enrich in pharyngeal and nerve ring BMs when secreted from distant sources, we fused *C. elegans* agrin with the fluorophore mScarlet-I ( *agr-1::mScarlet-I* ) and drove its expression from three different tissue-specific promoters: *myo-3* (body wall muscle), *pept-1* (intestine), and *rab-3* (pan-neuronal) ( **Figure 1A, B** ); (Benner et al., 2011; Bindels et al., 2017; Okkema et al., 1993; Silva-Garcia et al., 2019; Stefanakis et al., 2015). Confocal imaging of bleach synchronized L4 larvae showed that AGR-1::mScarlet-I driven from all three promoters resulted in mScarlet-I signal in the extracellular fluid located between tissues ( **Figure 1C** ; n = 8/10 animals or more for all promoters compared with 5/10 for endogenous AGR-1::mNG) and in BMs ( **Figure 1D** ). The highest levels of extracellular AGR-1::mScarlet-I were driven by *myo-3* (body wall muscle, average fluorescence a.u. = 78) and *pept-1* (intestine, a.u. = 71.), and lower levels were present when driven by *rab-3* (pan-neuronal, a.u. = 20). Interestingly, we also observed many internalized AGR-1::mScarlet-I puncta in the pharyngeal tissue, which were largely absent in animals where agrin was endogenously tagged with mNG ( **Figure 1E, G** ). We next quantified AGR-1::mScarlet-I and endogenous AGR-1::mNG localization at the pharyngeal BM and nerve ring BM ( **Figure 1B, D** **and** **F** ). Secretion from all three tissues (body wall muscle, intestine and neuron) resulted in strong enrichment of AGR-1::mScarlet-I in the pharyngeal BM with the greatest enrichment in the anterior pharyngeal bulb, similar to endogenous AGR-1::mNG (n = 10/10 animals for each, **Figure 1D** ). This suggests that the pharyngeal BM, and especially the anterior bulb, harbors high affinity binding sites for agrin. In contrast to the pharynx, we only observed AGR-1::mScarlet-I localization to the nerve ring BM when its expression was driven from the neurons ( **Figure 1D** ). This suggests that agrin localization to the nerve ring BM is dependent on direct addition from the neuronal cells in contact with the BM.

We next examined two BMs where endogenous agrin is not detectable— the body wall muscle BM and intestinal BM ( **Figure 1D** ). When agrin was expressed from either the body wall muscle or intestinal promoters, we observed localization to both the body wall muscle and intestinal BMs. Notably, when expression was driven by either promoter, levels of AGR-1::mScarlet-I in both the intestinal and body wall muscle BMs were at least ~ threefold lower (ranged ~3 to 13-fold) than the pharyngeal BM. These observations indicate that the body wall muscle and intestinal BMs have significantly lower affinity for agrin when compared to the pharyngeal BM. Expression driven by the pan-neuronal promoter *rab-3* did not result in localization to either the intestinal or body wall muscle BMs. However, this might be because extracellular levels of agrin when driven by the *rab-3* promoter were not high enough for addition into these BMs.

Taken together, our studies suggest that the pharyngeal BM has a high affinity for binding agrin and is capable of incorporating exogenous agrin to a greater extent than BMs surrounding the producing tissues (body wall muscle and intestine). We hypothesize that the combination of the pharyngeal BMs high affinity for binding agrin (likely provided by other BM proteins and receptors) and endogenous expression and secretion of agrin from the bordering pharyngeal epithelium ensures high levels of agrin specifically in the pharyngeal BM. In contrast, exogenous agrin only localized to the nerve BM when expressed under the pan-neuronal promoter. This suggests that the nerve ring BM might have a lower affinity for binding agrin and thus requires high concentration of localized deposition. Alternatively, agrin might be modified post-translationally in neurons to allow specific affinity for the nerve ring BM. We also found that BMs where agrin is not normally localized still have the potential to harbor agrin albeit at a lower efficiency, as the muscle and intestinal driven expression of agrin resulted in low accumulation of agrin in these BMs. Finally, we noticed that AGR-1::mScarlet-I supplied by other tissues and added from the extracellular fluid to the pharyngeal BM was extensively internalized within the pharyngeal epithelial cells. This contrasts with endogenous AGR-1::mNG, of which very little was internalized. As AGR-1::mScarlet-I expressed in other cells is being added to the pharyngeal BM in addition to endogenous agrin in these experiments, the number of binding sites for agrin might be saturated and this could result in selective internalization of the excess agrin in this tissue. It is also possible that agrin generated in other tissues might not have the optimal post-translational modifications that allow the most stable integration into the pharyngeal BM, and thus might be more readily internalized and removed from the BM. Taken together, our studies indicate a generalized requirement for local agrin production to enable specific BM localization, and that high affinity binding for agrin might help ensure increased enrichment in the pharyngeal BM. 

## Methods

***C. elegans* culture:**

*C. elegans* were grown at 20°C on nematode growth medium plates containing OP50 *E.* coli according to standard procedures (Stiernagle, 2006). Strains used in this study are listed in Table 1.

**Table 1: *C. elegans* strains used in this study**

**Table:** 

**Strain Name**	**Genotype**
N2	Wild-type (ancestral)
NK2353	qy27 [ agr-1 :: mNeonGreen]
NK3627	*qy379* [ *rab-3 p:: agr-1 ::mScarlet-I* ] I
NK3628	*qy380* [ *myo-3 p:: agr-1 ::mScarlet-I* ] I
NK3663	*qy387* [ *pept-1 p:: agr-1 ::mScarlet-I* ] I

**Generation of transgenic constructs:**

The regulatory sequences upstream of *myo-3* (~2.4kb), *pept-1* (~2.1kb) and *rab-3 b (~800bp)* were amplified from wild-type genomic DNA. Full length agrin cDNA was amplified from wild-type cDNA. Each promoter and agrin product pair was Gibson assembled into a plasmid containing the pAP088 backbone, *mScarlet-I* gene, *unc-54* 3' UTR, and homology arms for the *ttTi4348* site using primers listed in Table 2 (Bindels et al., 2017; Pani and Goldstein, 2018).

**Table 2: Gibson primers used in this study**

**Table:** 

**Identifier**			**Sequence (5' → 3')**
**Overhang**	**Target**	**Direction**	
088 backbone	*pept-1* promoter	Forward	GATCATCCtgtaaaacgacggccagtaaaGCTAGCaggccgaagaggtcctggaaaaaacc
*agr-1* cDNA	*pept-1* promoter	Reverse	gctcattcctcgtcagtatcgccactGGGCCCaaaATGAGACTTCACAATTATTTCGTTTC
*mScarlet-I*	*agr-1* cDNA	Reverse	GGAGACCATcgATGCTCCTGAGGCTCCCGATGCTCCGTTATAGCAGTACTTGGGTGTCTCT
088 backbone	*myo-3* promoter	Forward	GTCGATCATCCtgtaaaacgacggccagtaaaGCTAGCtctgttttcgttaattttgaat
*agr-1* cDNA	*myo-3* promoter	Reverse	AACGAAATAATTGTGAAGTCTCATtttGGGCCCatttctagatggatctagtggtcgt
088 backbone	*rab-3* promoter	Forward	CATCCtgtaaaacgacggccagtaaaGCTAGCattatctaacacaatgagaataa
*agr-1* cDNA	*rab-3* promoter	Reverse	ACGAAATAATTGTGAAGTCTCATtttGGGCCCcatctgaaaatagggctactgta

**Strain generation**

Strains were generated using CRISPR/Cas9-mediated homologous recombination using a self-excising cassette (SEC) for drug selection (hygromycin) to insert the transgenes into the *ttTi4348* transposon insertion site on chromosome I as described by (Dickinson and Goldstein, 2016; Frokjaer-Jensen et al., 2012; Keeley et al., 2020). The pCFJ352 plasmid containing the sgRNA sequence was used to direct Cas9 cleavage near this region for insertion.

**Microscopy**

Confocal imaging was performed at 20°C on a Zeiss Axio Imager A1 microscope fitted with a Yokogawa CSU-W1 spinning disk and an ORCA-Fusion CMOS camera using 505-, and 561-nm excitation lasers and a Zeiss 40× Plan Apochromat (1.4 numerical aperture) oil-immersion objective. All microscopes were controlled with μManager software v2.0.1 (Edelstein et al., 2010). Bleach synchronized L4 larvae were mounted on 5% noble agar pads with 0.01 M sodium azide (Kelley et al., 2017).

**Quantification of Agrin BM localization**

Single Z-slice confocal images were quantified using Fiji (V2.18.0). BM fluorescence intensity was measured by drawing a ~0.3µm wide and approximately 8-10µm long line along the BM of interest and recording the mean fluorescence value. Pharyngeal BM measurements were recorded from the BM surrounding the anterior bulb at a slice bisecting the animal where the pharynx was clearly visible. Both body wall muscle and nerve ring BM measurements were recorded at a slice where the isthmus of the pharynx began to appear. Body wall muscle BM measurements were taken at a region of the body wall muscle adjacent to the isthmus of the pharynx. Nerve ring BM measurements were recorded along the nerve ring. To account for autofluorescence, background measurements were acquired by drawing a line either next to the pharynx (for pharyngeal, body wall muscle, and nerve ring BM measurements) or next to the intestine (for intestine BM measurements) where no agrin signal was visible and subtracted from the raw fluorescence values measured at the BM (n=10).

**Quantification of Agrin Internalization puncta**

Internalization of agrin signal was measured in the anterior bulb of the pharynx at a slice bisecting the animal where the pharynx was clearly visible (n=10). Puncta were quantified by drawing a ~ 50μm ^2^ box in the center of the anterior bulb and recording the number of distinct puncta contained within.

**Quantification of Agrin extracellular fluorescence intensity**

Single Z-slice confocal images were quantified using Fiji (V2.18.0). Agrin extracellular fluorescence intensity was measured by drawing a ~0.3µm wide and approximately 8-10µm long line at the extracellular region between the pharynx and the intestine and recording the mean fluorescence value. Measurements were recorded at a slice bisecting the animal where the pharynx and intestine were clearly visible (n=10). To account for autofluorescence, background measurements were acquired by drawing a line either next to the pharynx where no extracellular agrin signal was visible and subtracted from the mean fluorescence values.

**Statistical Analysis**

Statistical analysis was performed in GraphPad Prism 10. The distribution of data was assessed for normality using the Kolmogorov–Smirnov test. To compare the number of agrin internalization puncta across the four strains, we used a one-way ANOVA followed by Tukey's multiple comparisons test for normally distributed data with equal variance.
